# Comprehensive analysis of KLHL35 expression and its prognostic value in cancer: implications for colorectal cancer diagnosis and therapy

**DOI:** 10.1007/s12672-025-03715-5

**Published:** 2025-10-20

**Authors:** Rong Qin, Yiyao Duan, Shasha Bao, Hui Wang, Jing Zhou, Xirui Fan, Xiang Li, Guoyu Li, Jun Hu

**Affiliations:** 1 Department of Gastroenterology, Yan’ an Hospital Affiliated to Kunming Medical University, Kunming, 650051 Yunnan China; 2 Key Laboratory of Tumor Immunological Prevention and Treatment of Yunnan Province, Kunming, 650051 China; 3 Department of Radiology, Yan’ an Hospital Affiliated to Kunming Medical University, Kunming, 650051 Yunnan China; 4 Department of Stomatology, Yan’ an Hospital Affiliated to Kunming Medica l University, Kunming, 650051 Yunnan China; 5 College of Basic Medical Sciences, Kunming Medical University, Kunming, 650500 Yunnan China; 6 Department of Colorectal Surgery, Yunnan Cancer Hospital, Yunnan, 650106 China; 7 The First People’s Hospital of Kunming, Yunnan, 650034 China

**Keywords:** KLHL35, Colorectal cancer, Prognostic biomarker, Cancer progression, Therapeutic target

## Abstract

This study investigates the expression patterns and clinical relevance of KLHL35 (Kelch-Like Family Member 35) across various cancer types, with a focus on colorectal cancer. Comprehensive analysis of RNA sequencing data from The Cancer Genome Atlas (TCGA) and protein expression data from the Human Protein Atlas (HPA) revealed significant overexpression of KLHL35 in 13 tumor types, including colorectal cancer. Increased KLHL35 expression was associated with poor clinical outcomes, including overall survival (OS), progression-free interval (PFI), and disease-specific survival (DSS). Receiver operating characteristic (ROC) curve analysis suggested promising but exploratory diagnostic value for KLHL35, with area under the curve (AUC) values exceeding 0.9 in colorectal cancer. Functional enrichment analyses suggested potential associations between KLHL35 and cell cycle/apoptosis pathways, while immune infiltration correlations hinted at a possible role in immune microenvironment remodeling. In vitro assays confirmed KLHL35’s role in promoting cancer cell proliferation, invasion, and migration. These findings suggest that KLHL35 could serve as a valuable diagnostic and prognostic biomarker and a potential therapeutic target, particularly in colorectal cancer. While these findings nominate KLHL35 as a candidate for therapeutic targeting, further mechanistic studies are required to validate its function.”

**Supplementary Information:**

The online version contains supplementary material available at 10.1007/s12672-025-03715-5.

## Introduction

Cancer remains one of the leading causes of mortality worldwide, with its intricate biological processes posing substantial challenges for diagnosis, treatment, and prognosis [1]. The heterogeneity of malignant tumors is reflected in various aspects, including the diversity of gene expression and the complexity of the tumor microenvironment across different cancer types [2]. This heterogeneity not only limits the efficacy of conventional treatments but also elevates the risk of recurrence and further complicates therapeutic strategies in various cancers [3, 4]. In recent years, advancements in molecular biology, genomics, and bioinformatics have significantly contributed to understanding the molecular mechanisms underlying tumor initiation and progression, enabling the identification of novel molecular markers driving personalized diagnostic and therapeutic strategies [5, 6]. The evolution of cancer therapeutics — from conventional cytotoxic agents to targeted therapies and immunomodulation — has been driven by deepening molecular insights, as comprehensively reviewed by Sonkin, who emphasize the pivotal role of multi-omics profiling in redefining treatment paradigms [7]. Central to this progress is The Cancer Genome Atlas (TCGA), which has enabled systematic dissection of molecular vulnerabilities across 33 tumor types through integrated genomic, transcriptomic, and proteomic data48. Seminal TCGA-based studies illustrate its translational impact: in breast cancer, immune profiling revealed exhausted CD8⁺ T-cell markers informing prognosis [8]; in glioma, CDK2, IGFBPs, and SCN3B emerged as actionable targets [9]; and in head-neck cancer, CNIH4 and AIMP1 functioned as auxiliary prognostic indicators [10, 11]; These findings collectively underscore how TCGA empowers the discovery of context-specific biomarkers and therapeutic niches.

KLHL35 (Kelch-Like Family Member 35) is a protein implicated in several critical cellular processes, including protein degradation, ubiquitination, and cell cycle regulation, which are fundamental for tumor cell survival and proliferation across multiple cancer types [12, 13]. Aberrant expression of KLHL35 has been associated with increased tumor cell proliferation, enhanced resistance to apoptosis, and therapeutic resistance. KLHL35 primarily exerts its effects by modulating the ubiquitin-proteasome pathway, influencing key processes such as protein turnover and cellular signaling. Additionally, KLHL35 interacts with various molecular complexes that govern cell growth and apoptosis, contributing to tumor development and progression. KLHL35 is also involved in the regulation of crucial signaling pathways related to tumor suppressor mechanisms and cell cycle control, highlighting its critical role in the broader tumorigenic process across cancers [14, 15]. Notably, while KLHL35 dysregulation has been reported in isolated cancer types, its pan-cancer significance—particularly as an immune-modulatory hub bridging sphingolipid metabolism and T-cell suppression—remains unexplored, representing a key knowledge gap. The rationale for prioritizing colorectal cancer (CRC) stems from three evidence-based observations: (1) CRC exhibits exceptionally high KLHL35 overexpression with strong diagnostic power; (2) Preliminary reports link KLHL35 to CRC immunosuppression; (3) CRC’s rising global burden (2 M new cases/year) demands novel therapeutic targets. Thus, CRC serves as an ideal model to dissect KLHL35’s context-specific mechanisms.

The objective of this study is to systematically analyze the expression patterns of KLHL35 across various cancer types using bioinformatics tools and publicly available databases. Additionally, this study aims to investigate the association between KLHL35 expression and tumor subtypes, clinical prognosis, and related molecular pathways. RNA sequencing data from resources such as TCGA and CCLE will be analyzed to evaluate KLHL35 expression in both tumor and normal tissues. Immunohistochemistry (IHC) will be employed to assess protein expression levels across different cancer types, while survival analysis will be performed to explore the relationship between KLHL35 expression and patient outcomes. Gene Ontology (GO) and KEGG pathway enrichment analyses will be used to identify the biological processes and signaling pathways associated with KLHL35. By integrating multiple data sources and experimental techniques, this research seeks to elucidate the role of KLHL35 in tumor biology and its potential applications in cancer diagnosis and therapy.

## Methods

### Analysis of KLHL35 expression in tissues and cells

RNA sequencing (RNAseq) data from 33 tumor types were sourced from the TCGA database (https://portal.gdc.cancer.gov) and processed utilizing the STAR pipeline. The data were extracted in TPM format, with matched tumor and adjacent normal tissue samples treated as paired datasets. All analyses were conducted using R software (version 4.2.1), employing packages such as ggplot2 [3.3.6], stats [4.2.1], and car [3.1-0]. The RNA expression data were transformed using log2(value + 1) before statistical evaluation. The Wilcoxon rank-sum and Wilcoxon signed-rank tests were applied for statistical comparisons. Data for cancer cell lines were obtained from the Cancer Cell Line Encyclopedia (CCLE), while data for normal cell lines were retrieved from the BioGPS database (http://biogps.org). To ensure cross-database comparability, rigorous harmonization strategies were implemented: (1) Normalization: All RNAseq data (TCGA, CCLE, BioGPS) were uniformly processed in TPM format. Database-specific biases were mitigated by quantile normalization using the preprocessCore R package [1.58.0], aligning expression distributions across platforms. (2) Batch correction: Technical batch effects (e.g., sequencing center, library preparation) were adjusted via the ComBat algorithm in the sva package [3.46.0], using database origin and project ID as covariates. This ensured comparability between TCGA tumor-normal pairs and CCLE/BioGPS cell line data.

### Analysis of KLHL35 protein expression in tissues

To investigate KLHL35 protein expression levels, immunohistochemistry (IHC) images were downloaded from the Human Protein Atlas (HPA) (http://www.proteinatlas.org), comparing normal tissues with tumor samples. This analysis covered various cancer types, including Colorectal Cancer, Lung Cancer, Head and Neck Cancer, Thyroid Cancer and Glioma. All images underwent HPA’s intrinsic quality control protocols, including antibody validation against recombinant proteins and siRNA knockdown controls, as well as pathology scoring by board-certified pathologists using predefined criteria (available at HPA Quality Documentation). Sample origins are documented in HPA’s metadata repository.

### KLHL35 expression in molecular and immune subtypes of tumors

The correlation between KLHL35 expression and different molecular and immune subtypes across various cancer types was evaluated using the TISIDB database (http://cis.hku.hk/TISIDB/). Expression data for KLHL35 in distinct molecular and immune subtypes were downloaded from the database, and subsequent correlation analyses were performed to assess the relationship between KLHL35 expression and specific cancer subtypes.

### Protein-protein interaction (PPI) network construction

To identify proteins potentially interacting with KLHL35 protein, we utilized the STRING database (https://string-db.org/). The top 20 proteins with the highest interaction confidence scores were selected for further analysis. The protein-protein interaction network was then constructed and visualized using Cytoscape software (version 3.9.0).

### Gene ontology (GO) and Kyoto encyclopedia of genes and genomes (KEGG) enrichment analyses

GO and KEGG pathway enrichment analyses were performed using the clusterProfiler package [4.4.4] following ID conversion of the gene list via the org.Hs.eg.db R package. This allowed for the investigation of biological processes, cellular components, and molecular functions associated with KLHL35 and its related genes.

### Diagnostic value assessment

The diagnostic significance of KLHL35 across multiple cancer types was assessed using receiver operating characteristic (ROC) curve analysis. Pan-cancer RNAseq data were obtained from the TCGA database, and samples without relevant clinical data were excluded from the analysis. ROC analysis was performed using the pROC package [1.18.0], and results were visualized with ggplot2 [3.3.6]. ROC analysis was performed using TCGA data, a widely accepted discovery cohort for initial biomarker screening. Diagnostic performance metrics are reported with the understanding that further validation is essential.

### Survival prognosis analysis

Kaplan-Meier survival curves were generated to evaluate the association between KLHL35 expression and cancer prognosis, including overall survival (OS), progression-free interval (PFI), and disease-free survival (DFS). Proportional hazards hypothesis testing and survival regression analyses were conducted using the survival package [3.3.1], with visualization performed using the survminer package [0.4.9] and ggplot2 [3.3.6]. Cox proportional hazards regression was applied, and significance was set at *p* < 0.05.

### Correlation of KLHL35 expression with clinical features in colorectal cancer (COAD)

RNAseq data from the TCGA-COAD project were processed in TPM format and linked with corresponding clinical data. Cox proportional hazards regression was utilized to assess the relationship between KLHL35 expression and clinical variables. Significant factors were subsequently included in a multivariate Cox model.

### Survival prediction models

To predict patient survival, a nomogram was created incorporating clinicopathological factors such as age, sex, cancer stage, disease type, smoking status, alcohol consumption, body mass index (BMI), and International Respiratory Symptom Score (IRSS). This model was constructed using the rms package. The predictive accuracy of the model was evaluated using ROC curves. LASSO (least absolute shrinkage and selection operator) regression was employed to reduce overfitting and improve model interpretability. Additionally, univariate Cox regression identified nine genes associated with colorectal cancer prognosis, which were included in the final risk factor model.

### Cell culture

Normal intestinal epithelial cells (NCM460) were obtained from Haixing Biosciences (China; Cat# TCH-C450), a commercial provider of authenticated cell lines. This spontaneously immortalized, non-transformed epithelial cell line originated from normal colonic mucosa and was confirmed via STR profiling. Colorectal cancer cell lines SW620 (Fuheng Biology, China; Cat# FH0021; ATCC reference: CCL-227) and RKO (Shanghai Chunmai Biotechnology, China; Cat# SHCY1773; ATCC reference: CRL-2577) were procured from authorized distributors, with STR authentication and functional validation (e.g., tumorigenicity in nude mice) provided by suppliers. All cell lines were tested for mycoplasma contamination prior to use. Cells were cultured in DMEM supplemented with 10% fetal bovine serum (FBS) at 37 °C in a 5% CO₂ incubator. When adherent cells reached 80%-90% confluence, they were trypsinized, counted, and replated. Suspended cells were centrifuged, resuspended, and plated in fresh medium.

### Western blotting (WB)

Cell lysates were prepared using RIPA buffer supplemented with 1% PMSF. Protein concentrations in the samples were quantified using the BCA assay. Proteins were resolved by 7.5% SDS-PAGE, transferred onto PVDF membranes, and incubated with the appropriate primary and secondary antibodies. After exposure, protein bands were visualized using a developer.

### In vitro cellular assays

Cells at 40%-60% confluence were transfected with lentiviruses for either knockdown (KD) or overexpression (OE) of KLHL35 for 48 h. Transfection efficiency was confirmed using fluorescence microscopy and WB analysis. Cell proliferation was assessed using the Cell Counting Kit-8 (CCK8) assay at 0, 1, 3, and 5 days inSW620, SW620-KD-KLHL35, and SW620-OE-KLHL35 cells. For wound healing assays, cells were plated into Culture-Inserts, and scratch closure was observed at 0, 24, 48, and 72 h. Scratch width was measured and quantified using ImageJ software. For invasion assays, Transwell plates were used. Matrix gel was diluted at a 1:8 ratio, and 100 µl of diluted gel was added to the upper chamber, followed by incubation at 37 °C for 30 min. A cell suspension (5 × 10^4 cells) was added to the upper chamber, and 500 µl of medium containing 20% FBS was added to the lower chamber. After 48 h of incubation, non-invading cells were removed, and invading cells were fixed with 4% paraformaldehyde and stained with 0.1% crystal violet. The number of invaded cells was counted in six random microscopic fields. For colony formation assays, 1,000 cells per well were seeded into 6-well plates. After 14 days of culture, cells were fixed with 4% paraformaldehyde, stained with crystal violet, and colonies were counted under a microscope. All functional assays included three technical replicates per group and were repeated in three independent biological replicates. Statistical significance was evaluated using two-way ANOVA with Tukey’s post-hoc test (for time-series) or unpaired t-test (for endpoint comparisons), with *p* < 0.05 considered significant.

## Results

### Differential expression of KLHL35 in tumors and adjacent normal tissues

**Fig. 1:**
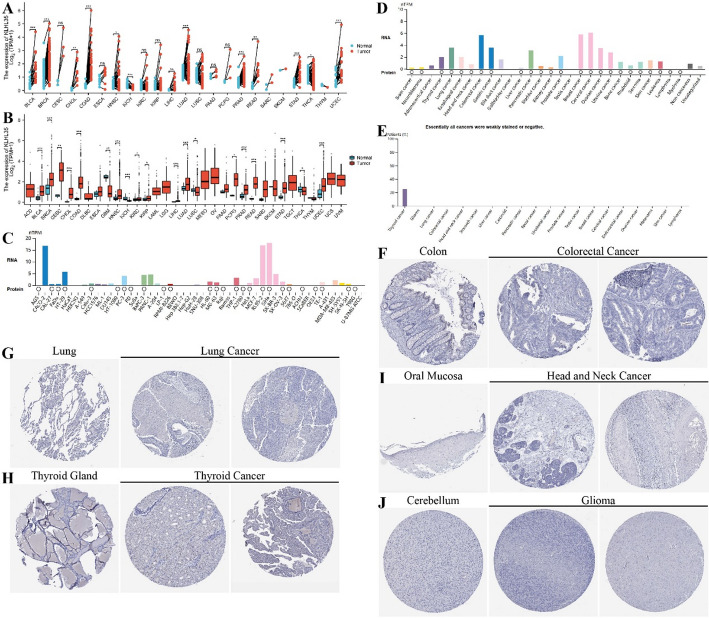
Differential analysis of KLHL35 expression in tumor and non-tumor tissues. **A**. KLHL35 mRNA expression in normal tissues and tumor cell lines (unpaired analysis). **B**. KLHL35 mRNA expression in normal tissues and tumor cell lines (paired analysis). **C**. Expression of KLHL35 mRNA and protein in cancer cell lines. **D**. KLHL35 mRNA and protein expression in tumor tissues. **E**. KLHL35 protein expression in tumor tissues from the HPA database. **F**. KLHL35 expression in normal colon tissues and colorectal cancer. **G**. KLHL35 expression in normal lung tissues and lung cancer. **H**. KLHL35 expression in the thyroid gland and thyroid cancer. **I**. KLHL35 expression in oral mucosa and head and neck cancer. **J**. KLHL35 expression in cerebellum and glioma. (*P < 0.05, **P < 0.01, ***P < 0.001)

Comprehensive analysis using the TCGA cancer database indicated that KLHL35 expression was significantly elevated in tumor tissues compared to adjacent normal tissues across 13 tumor types, including BLCA, BRCA, CHOL, COAD, and HNSC (Fig. 1A). Similarly, analysis of unpaired samples revealed differential expression in even more tumor types, covering 19 types such as BLCA, BRCA, CESC, CHOL, and COAD (Fig. 1B). Further investigation of KLHL35 levels across various cancer cell lines using the BioGPS database demonstrated moderate to high expression, with consistent mRNA and protein expression patterns (Fig. 1C). Additionally, we assessed KLHL35 protein levels across different cancer types and found that both mRNA and protein levels were significantly elevated in most cancers (Fig. 1D). Immunohistochemistry (IHC) results from the HPA database were compared with gene expression data, highlighting the top-ranking cancers in terms of KLHL35 protein expression, including Colorectal Cancer, Lung Cancer, Head and Neck Cancer, Thyroid Cancer and Glioma (Fig. 1E-J). These findings suggest that KLHL35 is highly expressed in tumor tissues and may be involved in tumorigenesis either directly or indirectly.

### Correlation between KLHL35 and molecular and immune subtypes in cancer

**Fig. 2:**
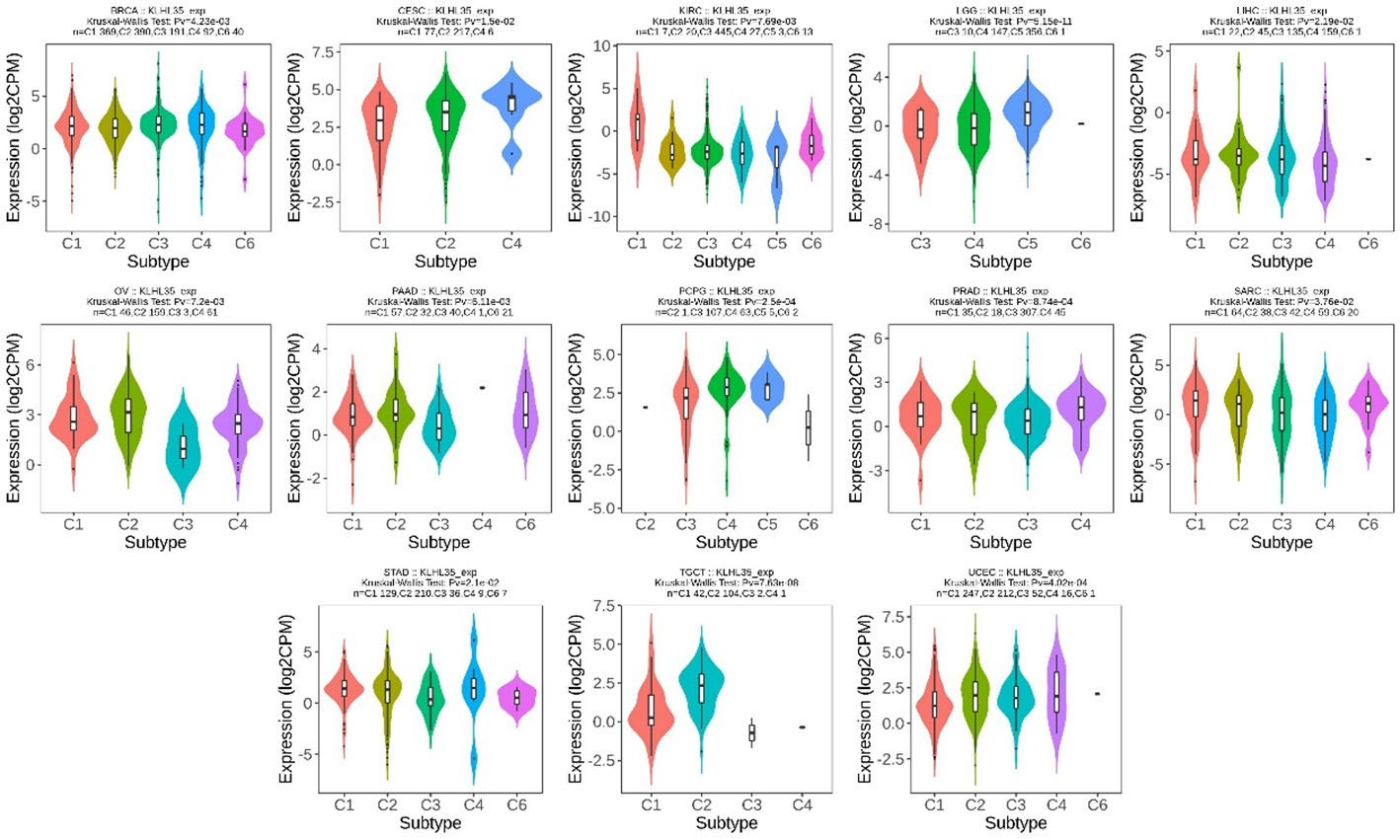
KLHL35 expression across different immune subtypes in pan-cancer

Using the TISIDB database, we discovered a significant association between KLHL35 expression and immune subtypes across multiple cancers. The immune subtypes included C1 (wound healing), C2 (IFN-gamma dominant), C3 (inflammatory), C4 (lymphocyte-depleted), C5 (immunologically quiet), and C6 (TGF-b dominant), with significant relevance observed in 13 cancers, such as BRCA, CESC, KIRC, LGG, LIHC, OV, PAAD, PCPG, PRAD, SARC, STAD, TGCT, and UCEC (Fig. 2, Supplementary Fig. 1). In most cancers, 4–5 distinct immune subtypes were present, suggesting a close relationship between KLHL35 and tumor immunity.

**Fig. 3:**
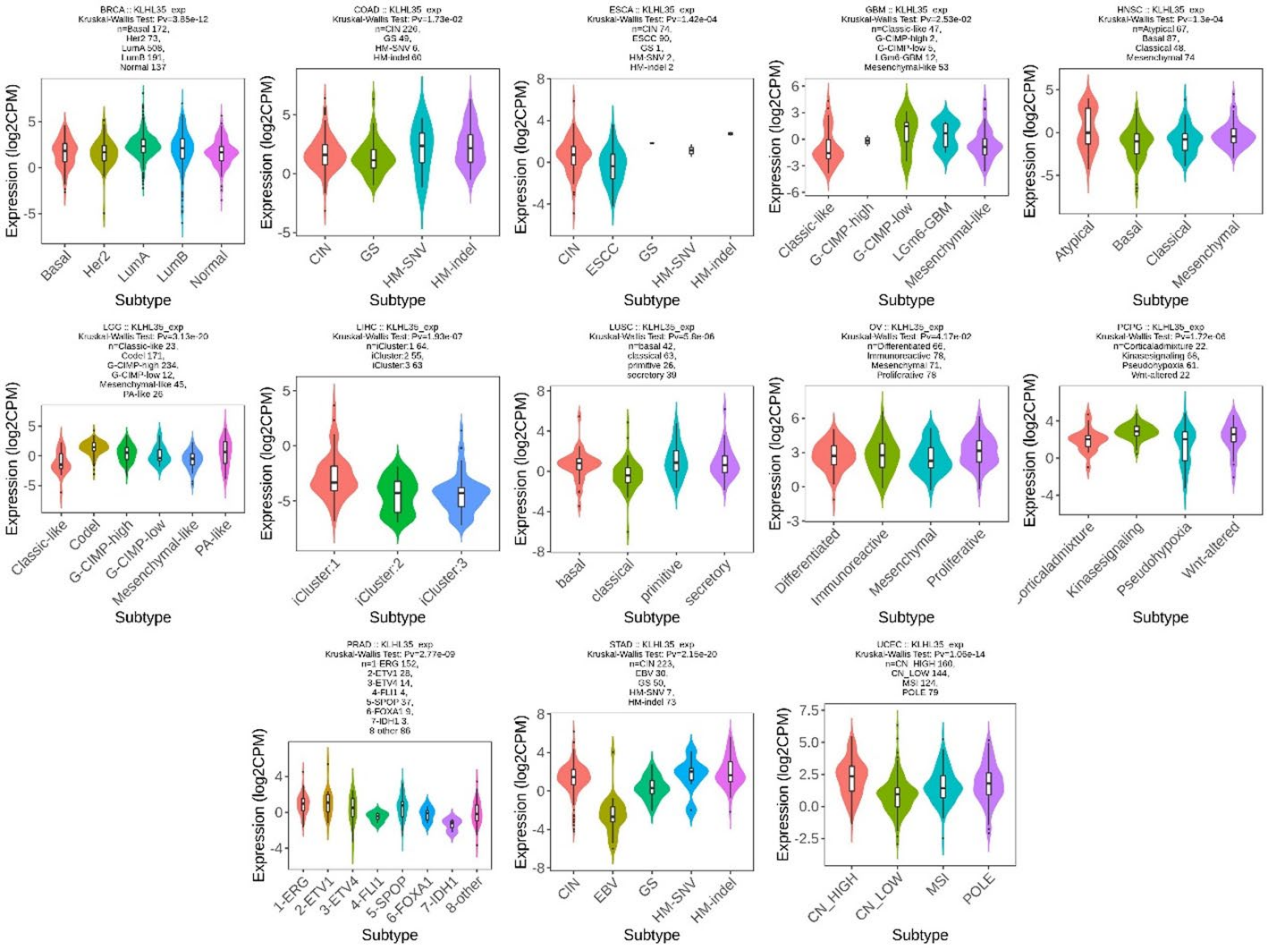
Analysis of KLHL35 molecular isoforms in pan-cancer

In addition, KLHL35 displayed differential expression across molecular subtypes in 13 cancer types, including BRCA, COAD, ESCA, GBM, HNSC, LGG, LIHC, LUSC, OV, PCPG, PRAD, STAD, and UCEC (Fig. 3, Supplementary Fig. 2). Most cancers showed 4–5 molecular subtypes, indicating a potential role for KLHL35 in cancer classification and treatment stratification. These observations could provide valuable insights for personalized cancer therapy.

### Diagnostic value of KLHL35 in Pan-Cancer analysis

**Fig. 4:**
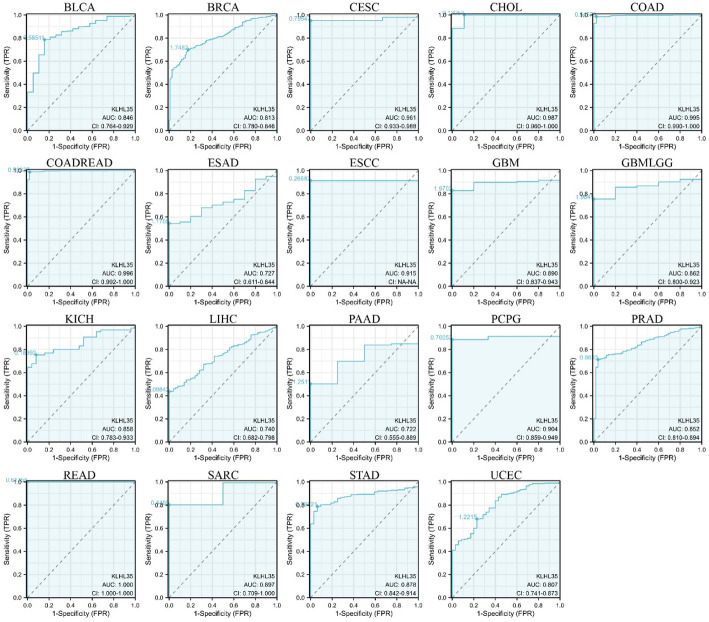
ROC curves evaluating KLHL35’s diagnostic performance across multiple cancer types

We evaluated the diagnostic potential of KLHL35 using ROC curves and found that KLHL35 demonstrated good diagnostic value in 24 cancers (Fig. 4, Supplementary Fig. 3). Notably, in 12 types, KLHL35 showed high accuracy (AUC > 0.7), including BLCA (AUC = 0.846), BRCA (AUC = 0.813), ESAD (AUC = 0.727), GBM (AUC = 0.890), GBMLGG (AUC = 0.862), KICH (AUC = 0.858), LIHC (AUC = 0.740), PAAD (AUC = 0.722), PRAD (AUC = 0.852), SARC (AUC = 0.897), STAD (AUC = 0.878), and UCEC (AUC = 0.807). In six cancers, KLHL35 demonstrated extremely high diagnostic performance (AUC > 0.9), including CESC (AUC = 0.961), CHOL (AUC = 0.987), COAD (AUC = 0.995), COADREAD (AUC = 0.996), ESCC (AUC = 0.915), and READ (AUC = 1.000). These findings highlight KLHL35 as a reliable biomarker for cancer diagnosis across multiple types.

### Prognostic value of KLHL35 in cancer

**Fig. 5:**
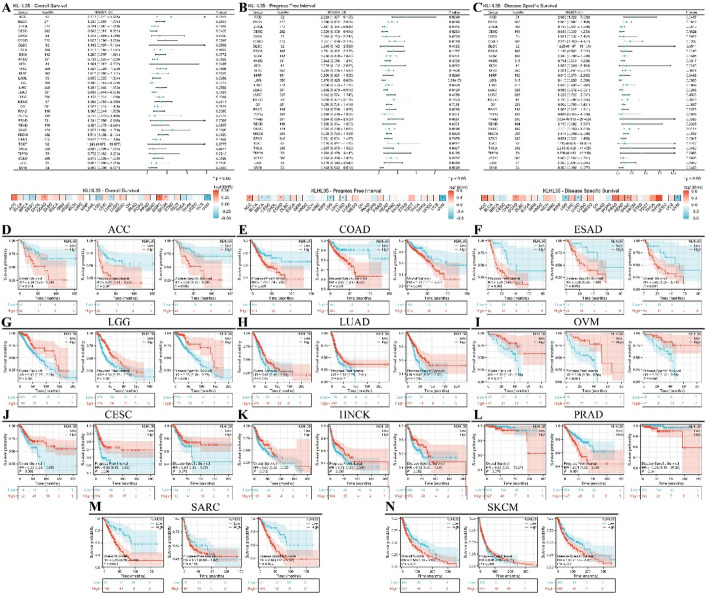
Prognostic and disease progression analysis of KLHL35 in pan-cancer. **A**–**C**. Forest plot and heatmap summarizing KLHL35’s prognostic value (OS, PFI, DSS). **D**–**N**. Kaplan-Meier (KM) curves illustrating KLHL35 expression and prognosis across various cancer types (ACC, COAD, ESAD, LGG, LUAD, OVM, CESC, HNSC, PRAD, SARC, SKCM)

Prognosis is a critical aspect of cancer patient management. We performed survival analyses based on KLHL35 expression levels in various cancers, examining overall survival (OS), progression-free interval (PFI), and disease-free survival (DFS) (Supplementary Fig. 4). Results, illustrated in a forest plot and heatmap (Fig. 5A), revealed that KLHL35 expression influenced OS, PFI, and DSS to varying extents in different cancers. KLHL35 had a positive prognostic impact in 10 cancers, with six cancers showing significant differences in all three survival metrics: ACC, COAD, ESAD, LGG, LUAD, and OVM. For instance, in ACC, OS (HR = 2.54, 95% CI 1.20–5.36, *P* = 0.0014), PFI (HR = 2.81, 95% CI 1.51–5.22, *P* = 0.001), and DSS (HR = 2.88, 95% CI 1.32–6.29, *P* = 0.008) were significantly affected by KLHL35 expression. Similarly, significant results were observed in COAD, ESAD, LGG, LUAD, and OVM. In contrast, KLHL35 overexpression was generally associated with poor prognosis in most cancers, indicating its potential role as an oncogenic factor.

### Analysis of KLHL35 Interaction/Associated proteins and downstream functional pathways

**Fig. 6:**
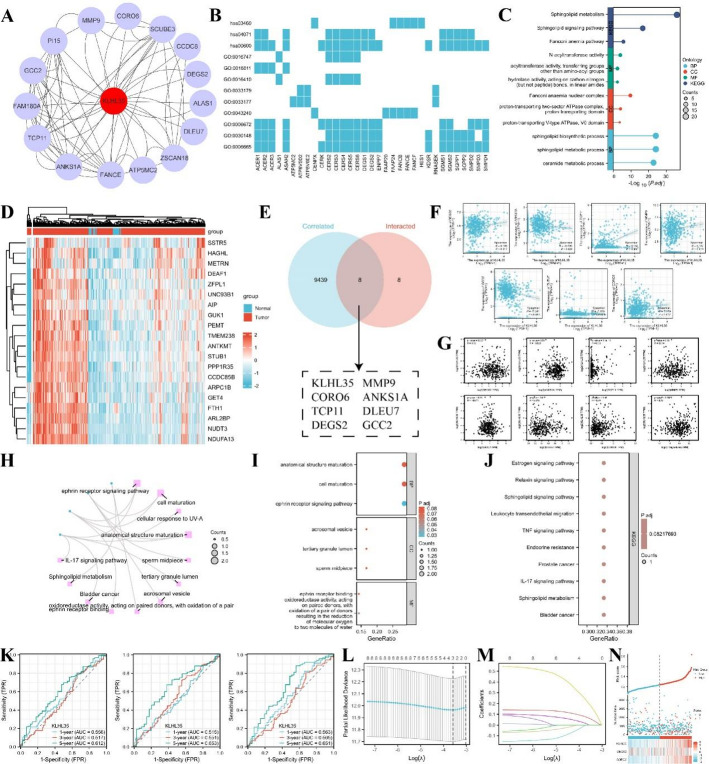
Correlation and enrichment analysis of KLHL35-related genes and proteins. **A**. Protein-protein interaction (PPI) network showing the top 20 proteins interacting with KLHL35. **B**, **C**. Heatmap and lollipop plot of GO and KEGG pathway analyses for KLHL35-related proteins. **D**. Heatmap of the top 30 genes correlated with KLHL35 in colorectal cancer. **E**. Venn diagram of overlapping genes/proteins. **F**, **G**. Eleven genes highly correlated with KLHL35. **H**. Network diagram of GO and KEGG pathways associated with KLHL35 and the 7 genes. **I**, **J**. Bubble plot of GO and KEGG analyses of KLHL35 and its interacting proteins. **K**. Time-dependent ROC curve predicting 1-, 3-, and 5-year survival. L-M. LASSO model results with cross-validation for tuning parameter selection and coefficient profiles. **N**. Risk score, survival status, and heatmap of eight genes in colorectal cancer patients

Using the STRING database and Cytoscape, we identified the top 15 proteins interacting with KLHL35 (Fig. 6A). GO enrichment analysis of these proteins revealed significant enrichment in biological processes such as sphingolipid metabolism, ceramide biosynthesis, and Fanconi anemia-related processes (Fig. 6B, C). Cellular components (CC) were primarily enriched in complexes such as the Fanconi anemia nuclear complex and proton-transporting ATPase complexes. Molecular functions (MF) included N-acyltransferase activity and hydrolase activity. KEGG pathway enrichment indicated associations with sphingolipid metabolism, Fanconi anemia pathway, and sphingolipid signaling (Fig. 6B, C).

To verify the role of KLHL35 in tumorigenesis, we focused on COAD, a tumor type where KLHL35 ranked highly across multiple analyses. Using datasets from colorectal cancer studies, we identified genes co-expressed with KLHL35, with the top 30 genes visualized in a heatmap (Fig. 6D, Supplementary Table 1). Further validation and interaction analysis using the STRING database identified eight proteins—KLHL35, CORO6, TCP11, DEGS2, MMP9, ANKS1A, DLEU7, and GCC2—as being significantly correlated with KLHL35 expression (Fig. 6G-I).

### Correlation between KLHL35 expression and immune cell infiltration in colorectal cancer

**Fig. 7:**
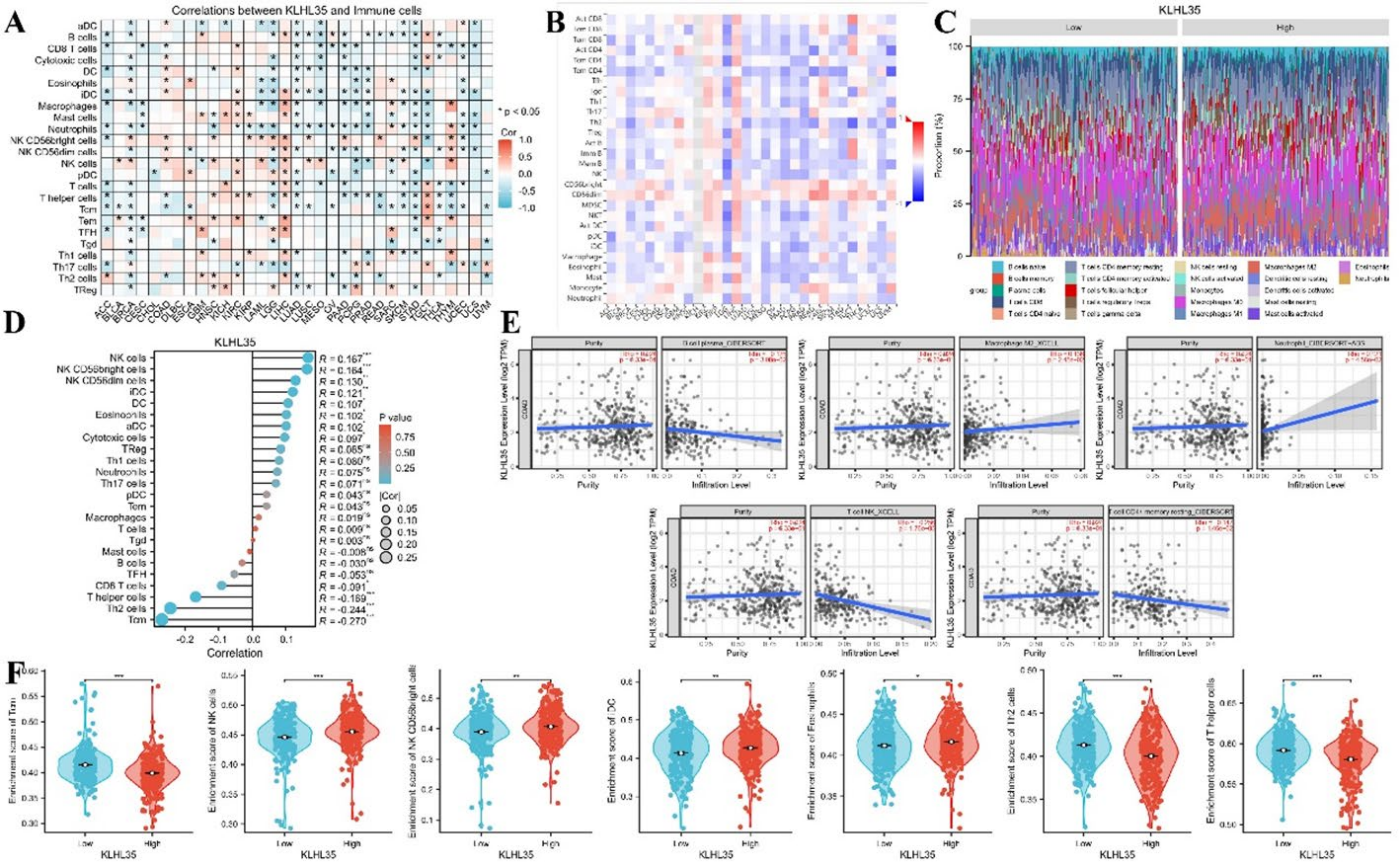
Association of KLHL35 expression with immune cell infiltration in cancer. **A**. Heatmap showing KLHL35 expression and immune cell infiltration in pan-cancer (TCGA). **B**. Heatmap showing KLHL35 expression and immune cell infiltration in pan-cancer (TISIDB). **C**. Box-overlap plot showing correlation between KLHL35 expression and immune cell types in colon cancer. **D**. Lollipop plot showing correlation between KLHL35 expression and immune cell types in colon cancer. **E**. Scatterplot showing correlation between KLHL35 expression and immune cell infiltration in colon cancer (TIMER2.0). **F**. Scatterplot showing correlation between KLHL35 expression and immune cell infiltration in colon cancer (TCGA)

Previous functional studies suggested a close association between KLHL35 and immune responses. We therefore examined the relationship between KLHL35 expression and the infiltration of 26 immune-related cell types. Heatmap results from a pan-cancer analysis indicated that Bioinformatic analyses suggested a potential association between high KLHL35 expression and immunosuppressive microenvironments. These database-derived observations generate the hypothesis that KLHL35 may facilitate immune evasion (Fig. 7A). Furthermore, validation of immune infiltration trends in the TISIDB database revealed consistent results with our downloaded data (Fig. 7B). Taking colorectal cancer as an example, we analyzed the specific correlation between KLHL35 and immune cell infiltration. Bar and lollipop charts demonstrated a positive correlation between KLHL35 expression and immune cell infiltration in colorectal cancer (Fig. 7C, D). TIMER2.0 database analysis confirmed significant correlations between KLHL35 and various immune cell types (Fig. 7E), such as B cells (Rho = -0.125), M2 macrophages (Rho = 0.136), neutrophils (Rho = 0.121), T cells (Rho = -0.256), and resting memory CD4 + T cells (Rho = -0.147). Finally, further analysis of TCGA data in colorectal cancer revealed significant correlations between KLHL35 and seven immune cell types, including Tcm, NK cells, NK CD56bright cells, iDC, eosinophils, Th2 cells, and T helper cells (Fig. 7F, Supplementary Fig. 5). These findings align with the pan-cancer analysis and indicate that KLHL35 is a potential target for cancer immunotherapy.

### Correlation between KLHL35 gene and clinical subgroups in colorectal cancer

**Fig. 8:**
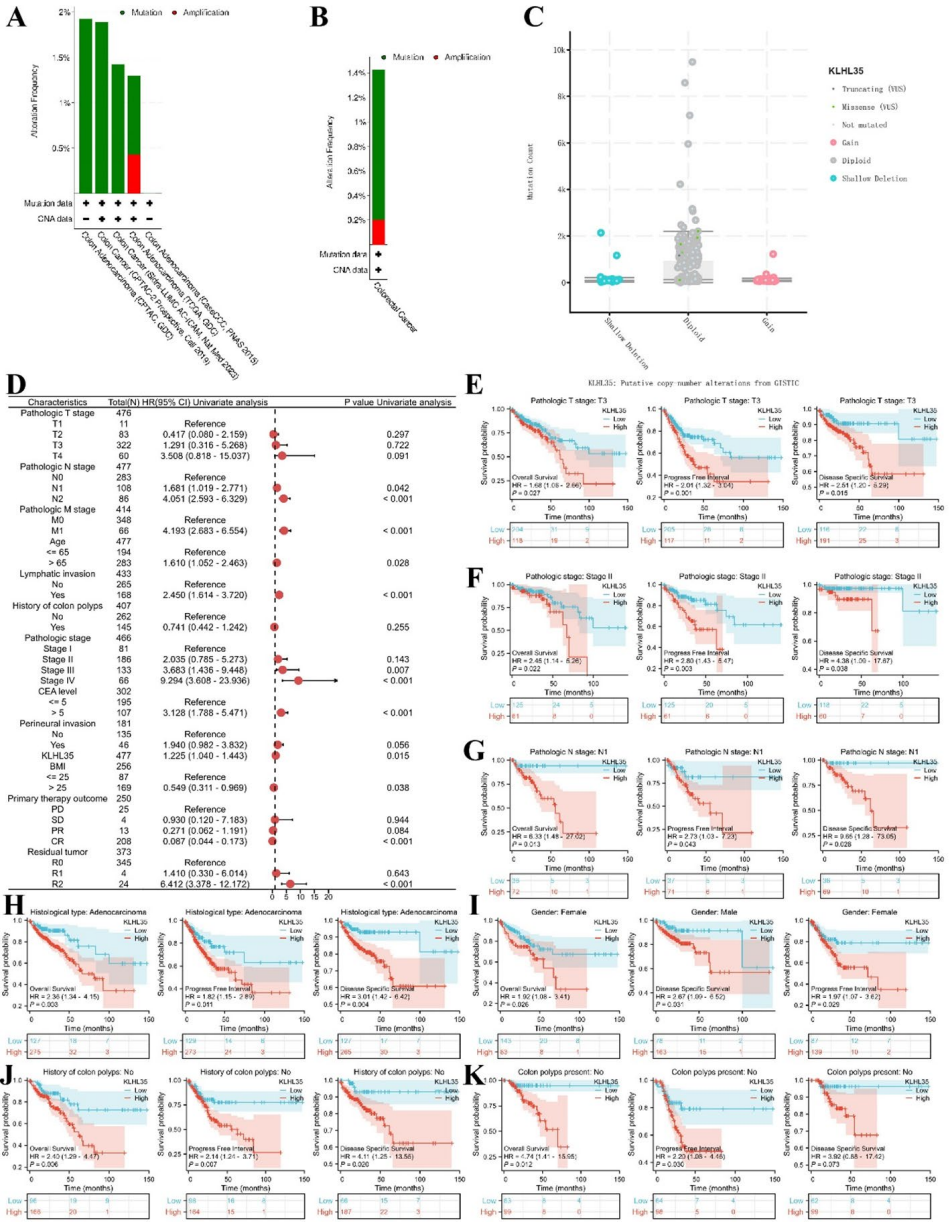
Genomic alterations of KLHL35 in colorectal cancer and their correlation with prognosis across clinical subgroups. **A**. OncoPrint of KLHL35 alterations in cancer cohorts. **B**. Breakdown of KLHL35 gene alterations in colorectal cancer. **C**. Major types of KLHL35 alterations. **D**. Forest plot summarizing KLHL35’s prognostic value in different clinical subgroups. **E**–**K**. Kaplan-Meier (KM) curves showing KLHL35 expression, prognosis, and disease progression in colorectal cancer, stratified by clinical factors

Using the cBioPortal database, we first explored genomic alterations in the KLHL35 gene in colorectal cancer and found that the frequency of KLHL35 genomic changes was less than 2% (Fig. 8A). Only two types of alterations led to changes in KLHL35 gene expression, with copy number variation (CNV) being a positive result in a few sequencing cases (Fig. 8B, C). Given the significant prognostic value of KLHL35 for OS, PFI, and DSS in colorectal cancer patients, we further investigated its role in different clinical features and patient subgroups. Results were presented as a forest plot (Fig. 8D, Supplementary Fig. 6, Supplementary Table 2), showing that KLHL35 had good prognostic significance across various clinical subgroups. For example, high KLHL35 expression in patients with pathologic T stage T3 was associated with poor prognosis, whereas its effect was not significant in other stages. Similarly, KLHL35 had the most significant impact on prognosis in stage II of the pathologic stage. Regarding pathologic N stage, KLHL35 had the most significant prognostic influence in stage N1. Among genders, KLHL35 had a more prominent prognostic impact in female patients compared to males. Most notably, patients without a prior history of colorectal disease who later developed colorectal cancer showed strong prognostic value for KLHL35, particularly since polyps are a high-risk factor for colorectal cancer. These results indicate that KLHL35 plays a pro-tumorigenic role in the prognosis and disease progression of colorectal cancer (Fig. 8E–J).

### Co-Expression genes and functional enrichment analysis of KLHL35 in colorectal cancer

**Fig. 9:**
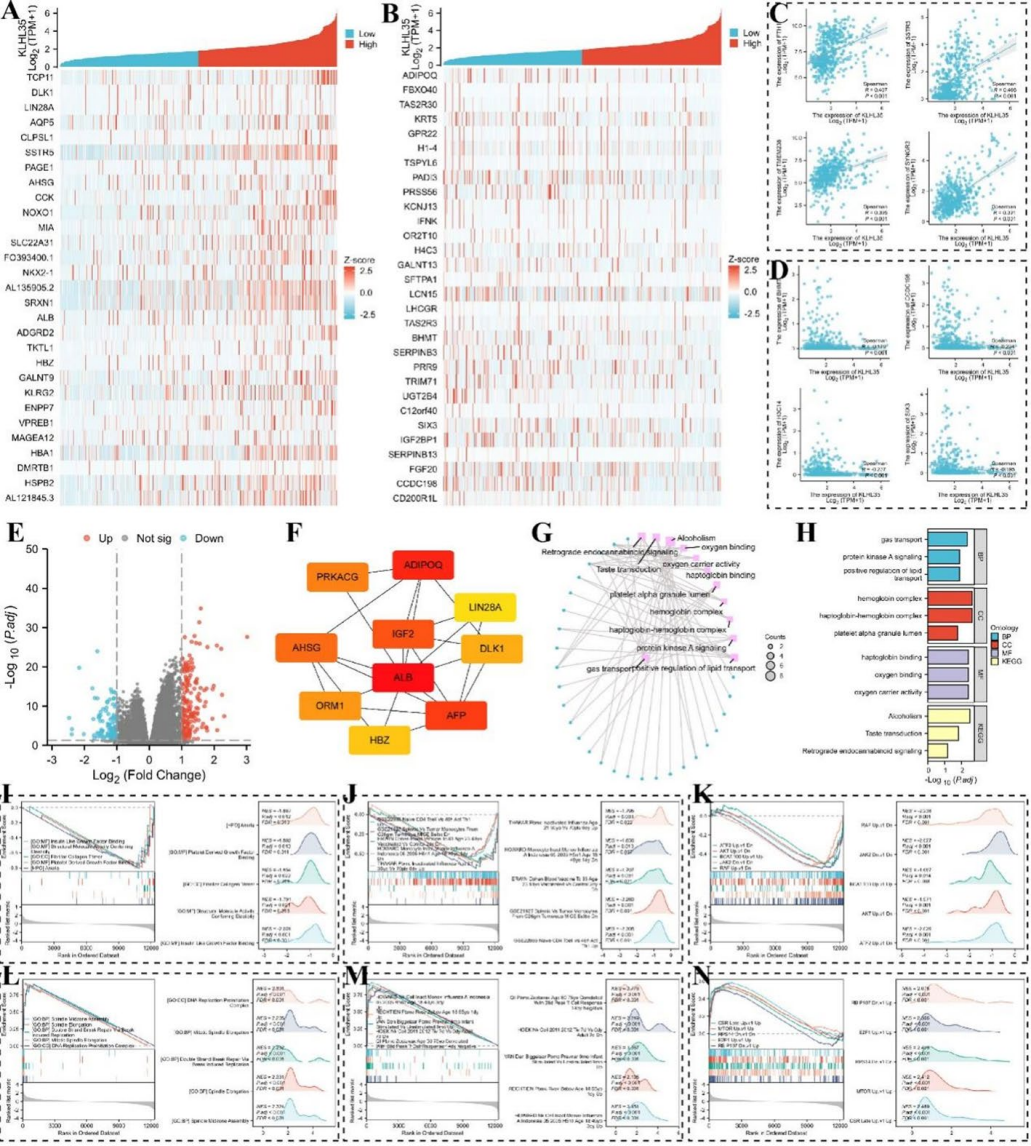
KLHL35 differential expression in colorectal cancer and functional enrichment analysis. **A**, **B**. Heatmaps of the top 30 genes positively and negatively correlated with KLHL35 in colorectal cancer. **C**, **D**. Scatterplot of the top 4 genes positively and negatively associated with KLHL35 in colorectal cancer. **E**, **F**. HUB genes correlated with KLHL35. **G**–**H**. Network diagram of GO/KEGG analyses of KLHL35 co-expressed genes. **I**–**N**. Visualization of GSEA results for KLHL35 co-expressed genes (Gene Ontology, immunological signatures, and oncogenic signatures)

We explored genes that were co-expressed with KLHL35 in colorectal cancer, displaying the top 30 positively and negatively correlated genes in a heatmap (Fig. 9A, B). Scatter plots showed the top four positively and negatively correlated genes (Fig. 9C, D). Using defined thresholds, we identified 1,233 differentially expressed genes (DEGs), including 118 upregulated genes and 39 downregulated genes (Fig. 9E, Supplementary Table 3). Protein-protein interaction (PPI) networks were constructed for the upregulated and downregulated genes, identifying key hub genes such as ALB, IGF2, ADIPOQ, AFP, AHSG, PRKACG, ORM1, DLK1, HBZ, and LIN28A (Fig. 9G). GO and KEGG enrichment analyses of DEGs indicated that KLHL35-related genes in colorectal cancer were involved in biological processes such as gas transport, protein kinase A signaling, and positive regulation of lipid transport. Cellular components enriched included hemoglobin complexes and platelet alpha granule lumens. Molecular functions were related to haptoglobin binding, oxygen binding, and oxygen carrier activity. KEGG pathways were primarily enriched in alcoholism, taste transduction, and retrograde endocannabinoid signaling (Fig. 9G–H).

Finally, we conducted GSEA enrichment analysis to explore the correlation of KLHL35-related DEGs with Gene Ontology (GO), immunological signatures, and oncogenic signatures. The top five positively and negatively correlated categories were presented in the figures (Supplementary Tables 4–6). GO analysis highlighted categories such as sister chromatid segregation, regulation of chromosome segregation, and metaphase-to-anaphase transition of the cell cycle (Fig. 9I, L). Immunological signatures were primarily associated with CD8 T cell activation and memory CD4 T cells (Fig. 9J, M). Oncogenic signatures were related to pathways like mTOR, E2F1, and RAF signaling (Fig. 9K, N). These results suggest that KLHL35 is closely associated with gene ontology, immunological, and oncogenic signatures, further supporting its role as a potential tumor biomarker and therapeutic target.

### KLHL35 is overexpressed in colorectal cancer cells and promotes malignant behavior

**Fig. 10:**
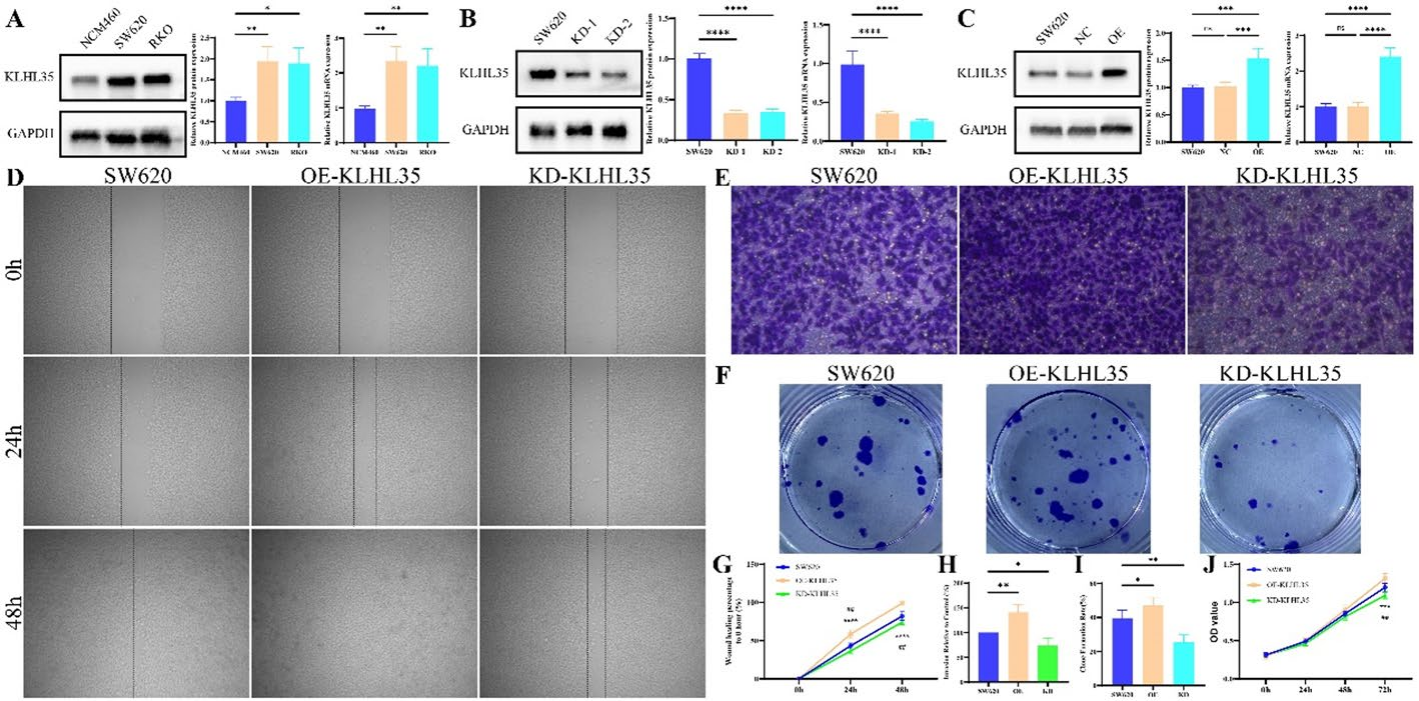
KLHL35 affects biological functions in colorectal cancer. **A**. Protein expression of KLHL35 in normal intestinal epithelial cells and two colorectal cancer cell lines. **B**. Western blot detection of KLHL35 knockdown after transfection with KLHL35-specific siRNA into SW620 cells. **C**. Western blot detection of KLHL35 overexpression after transfection with KLHL35-specific or control oeRNA into SW620 cells. **D**. Effect of KLHL35 on the migration ability of SW620 cells. **E**. Effect of KLHL35 on the invasion ability of SW620 cells. **F**. Effect of KLHL35 on the proliferative capacity of SW620 cells (plate colony formation assay). **G**. Statistical analysis of cell migration results. **H**. Statistical analysis of cell invasion results. **I**. Statistical analysis of cell proliferative capacity results (plate colony formation assay). **J**. Effect of KLHL35 on the proliferative capacity of SW620 cells (CCK-8 assay)

Western blot (WB) analysis demonstrated that KLHL35 mRNA and protein expression were significantly elevated in two colorectal cancer cell lines compared to normal intestinal epithelial cells (Fig. 10A). Gene overexpression and knockout experiments in the SW620 colorectal cancer cell line showed that KLHL35 expression could be effectively upregulated or inhibited (Fig. 10B-C). Inhibition of KLHL35 expression significantly reduced the invasion, migration, and proliferation of SW620 cells. Conversely, overexpression of KLHL35 in colorectal cancer cells significantly enhanced their proliferative, migratory, and invasive abilities (Fig. 10D-J).

## Discuss

This study provides a comprehensive investigation into the expression patterns and clinical relevance of KLHL35 across a wide range of cancers. Our findings indicate that KLHL35 levels are significantly elevated in multiple tumor types compared to adjacent normal tissues, with notable overexpression in 13 cancers, including colorectal cancer (COAD), bladder cancer (BLCA), breast cancer (BRCA), and head and neck squamous cell carcinoma (HNSC). Importantly, protein overexpression was confirmed through immunohistochemistry (IHC) analysis in several cancers, aligning with mRNA expression patterns. These results suggest that KLHL35 is highly expressed in tumor tissues and could play a pivotal role in tumorigenesis. ROC curve analysis revealed that KLHL35 has strong diagnostic potential, with area under the curve (AUC) values exceeding 0.9 in several cancer types, including colorectal cancer, indicating its potential as a biomarker for cancer diagnosis. Despite high AUCs (>0.9), these diagnostic metrics originate from a retrospective database (TCGA) and require confirmation in prospectively collected, multi-center cohorts to assess clinical utility [16]. Additionally, survival analysis demonstrated that high KLHL35 expression correlates with poorer overall survival (OS), progression-free interval (PFI), and disease-specific survival (DSS) in multiple cancers. For instance, in colorectal cancer, KLHL35 overexpression was associated with significantly worse outcomes, further emphasizing its role as an oncogenic factor.

As one of the first studies to systematically evaluate KLHL35 expression across various cancers and its relationship to clinical outcomes, this research confirms the potential of KLHL35 as a critical factor involved in tumor progression. The observed association between high KLHL35 expression and poor prognosis suggests that KLHL35 may influence cancer progression by promoting cell proliferation, inhibiting apoptosis, and facilitating immune evasion. These findings align with previous research that identified overexpression of KLHL35 in certain cancers as an indicator of aggressive tumor behavior and poor clinical outcomes. Our study expands this understanding by performing a pan-cancer analysis and investigating correlations with molecular and immune subtypes. KLHL35 was found to be significantly associated with various immune subtypes across multiple cancers, such as the IFN-gamma dominant and inflammatory subtypes, indicating a potential role in the immune microenvironment. These associations suggest that KLHL35 may have a role in modulating immune responses, which could have implications for cancer immunotherapy. Furthermore, the interaction between KLHL35 and immune cell infiltration, particularly in colorectal cancer, indicates that KLHL35 may contribute to immune suppression, thereby promoting tumor growth. As these immune correlations are derived solely from computational databases (TISIDB, TIMER2.0), future work should prioritize experimental validation through: (1) Multiplex immunohistochemistry (mIHC) quantifying KLHL35 expression with immune markers (CD8, FOXP3, PD-L1) in patient tissue microarrays. (2) Flow cytometry profiling of KLHL35-knockdown tumors in syngeneic mouse models. (3) Spatial transcriptomics to map KLHL35-immune cell interactions.”

The construction of the KLHL35 protein-protein interaction (PPI) network using the STRING database, alongside GO and KEGG enrichment analyses, provided further insights into the biological processes involving KLHL35. Our results indicate that KLHL35 is actively involved in pathways related to sphingolipid metabolism, Fanconi anemia, and cell cycle regulation, all of which contribute to tumor growth and survival. Additionally, KLHL35’s interaction with other proteins involved in apoptosis, cell cycle regulation, and DNA repair highlights its central role in tumor biology. Given KLHL35’s significant overexpression and poor prognostic implications, it holds great promise as a marker for cancer diagnosis and prognosis. Our results show that KLHL35’s prognostic impact is particularly pronounced in early-stage cancers, including colorectal cancer, indicating its potential utility in early cancer detection. Detecting KLHL35 levels in cancers such as colorectal cancer could aid in predicting patient outcomes and inform personalized treatment strategies. For instance, targeting KLHL35 through small molecule inhibitors or antibodies that disrupt its interaction with other proteins could potentially improve therapeutic efficacy, especially in combination with chemotherapy or immunotherapy [17–19].

While our study has highlighted the diagnostic and prognostic significance of KLHL35, several key questions remain unresolved, particularly regarding its mechanistic role in tumor biology. The exact pathways through which KLHL35 exerts its effects on cell proliferation, apoptosis inhibition, and immune regulation still require further elucidation. Addressing these gaps could pave the way for the development of more effective cancer therapies that target KLHL35. From a medical perspective, KLHL35’s overexpression in various cancer types opens the possibility of integrating KLHL35 testing into routine clinical diagnostics. Specifically, in colorectal cancer, KLHL35 could serve as a valuable biomarker for early detection, allowing clinicians to identify high-risk patients and tailor treatment strategies accordingly. Moreover, understanding the immune-modulatory role of KLHL35 could lead to breakthroughs in immunotherapy. As immunotherapies become a cornerstone in cancer treatment, targeting proteins like KLHL35, which may influence immune cell infiltration and immune evasion, could enhance the efficacy of existing therapies or lead to the development of new ones [20–22]. Targeting KLHL35 in clinical practice may leverage dual strategies informed by its dual roles: as a cell-autonomous oncoprotein and an immune microenvironment modulator. While no direct KLHL35 inhibitors currently exist, lessons from other Kelch-family targets suggest promising avenues. For instance, inhibitors against Keap1 (KLHL19) disrupt its binding to Nrf2, activating antioxidant responses in cancer [23]; similarly, KLHL20-targeting agents destabilize its interaction with PML/DAPK1 to suppress tumor survival [23]. Our immune subtype analysis (e.g., IFN-γ-dominant subtypes) positions KLHL35 as a candidate for combination immunotherapy—potentially enhancing checkpoint blockade by reversing its immunosuppressive function in T-cell/NK cell infiltration. To optimize therapeutic design, we can apply the dual-targeting framework, advocating concurrent disruption of tumor-intrinsic pathways (e.g., sphingolipid metabolism via KLHL35) and reactivation of immune surveillance [24]. Practically, this might involve bispecific antibodies (e.g., targeting KLHL35 and 4-1BB) or CRISPR-engineered CAR-T cells co-targeting KLHL35-related antigens—strategies that balance cell-directed and microenvironmental interventions per next-generation regimen principles.

In summary, KLHL35 emerges as a promising candidate for both cancer diagnosis and treatment. Its significant overexpression across multiple cancers, combined with its association with poor clinical outcomes, indicates its potential as a therapeutic target. While this study demonstrates KLHL35’s clinical significance, certain limitations should be acknowledged: Our findings primarily derive from bulk transcriptomic analyses of retrospective TCGA datasets, which carry inherent biases in sample composition (e.g., tumor heterogeneity, limited normal controls) and technical processing (e.g., batch effects, platform variability) that necessitate cautious interpretation of differential expression and pathway results [25]; furthermore, while IHC confirmed protein overexpression, mechanistic insights remain reliant on mRNA correlations with limited direct human model validation. Future efforts should validate these observations in prospective multi-center cohorts, employ single-cell sequencing to resolve cell-type-specific functions, establish CRISPR-engineered isogenic models to dissect KLHL35’s roles in sphingolipid metabolism and immune evasion, and test therapeutic targeting in patient-derived organoids or immunocompetent murine systems to bridge species-specific gaps.

## Supplementary Information

Below is the link to the electronic supplementary material.

Supplementary Material 1.

Supplementary Material 2.

Supplementary Material 3.

Supplementary Material 4.

Supplementary Material 5.

Supplementary Material 6.

Supplementary Material 7.

Supplementary Material 8.

Supplementary Material 9.

Supplementary Material 10.

Supplementary Material 11.

Supplementary Material 12.

## Data Availability

The datasets generated and analyzed during this study are available in the TCGA ( [https://portal.gdc.cancer.gov](http:/\/portal.gdc.cancer.gov) ), BioGPS database ( [http://biogps.org](http:/\/biogps.org) ) , TISIDB database ( [http://cis.hku.hk/TISIDB/](http:/\/cis.hku.hk/TISIDB) ), STRING database ( [https://string-db.org/](https:/\/string-db.org) ) and HPA ( [https://www.proteinatlas.org](https:/\/www.proteinatlas.org) ) repositories. Original Western blot images are provided in the Supplementary Material.
