# Supplementary figures and images for: Comprehensive analysis of KLHL35 expression and its prognostic value in cancer: implications for colorectal cancer diagnosis and therapy
Source: Discov Oncol. 2025 Oct 20;16:1921. doi: 10.1007/s12672-025-03715-5 (PMC12537653; doi:10.1007/s12672-025-03715-5)

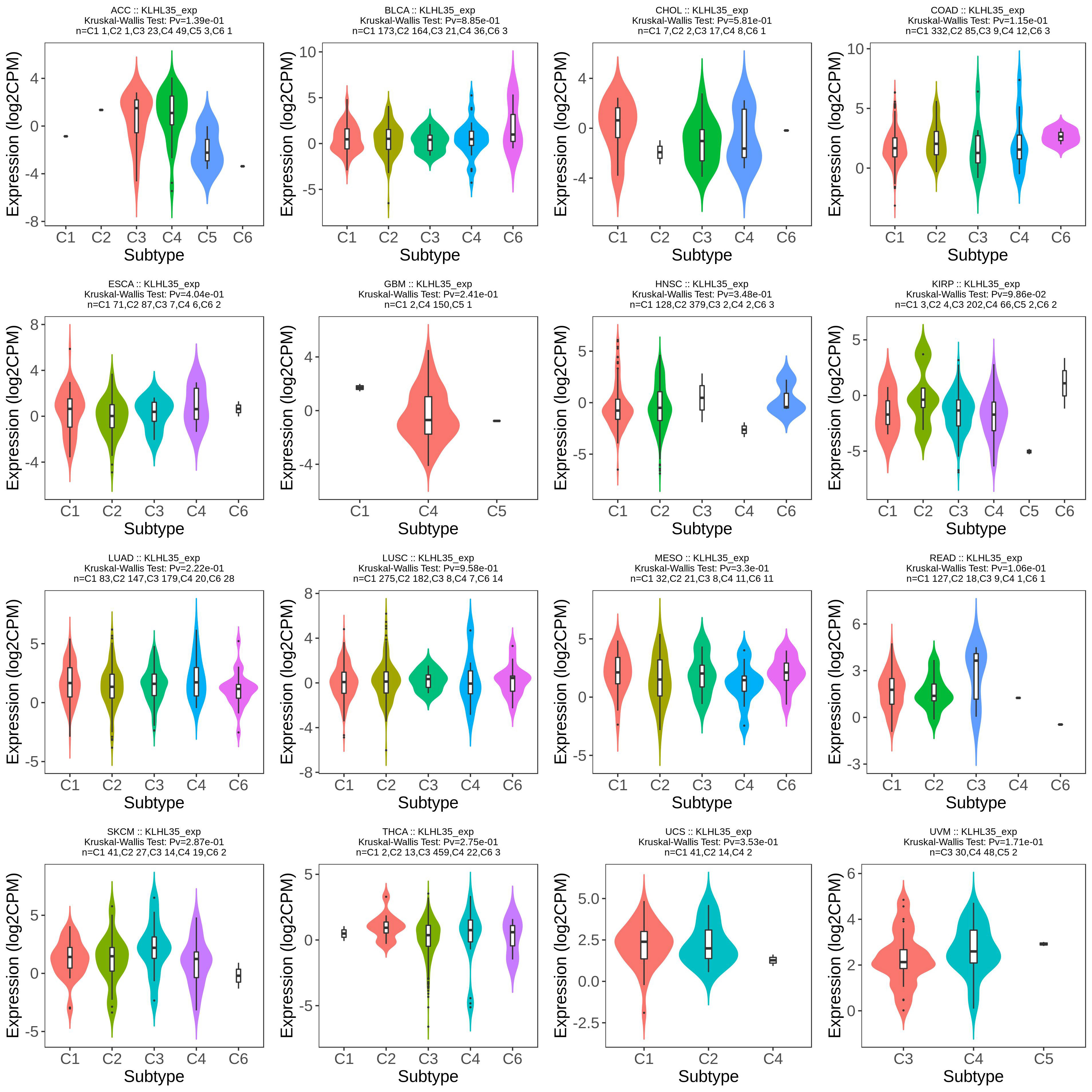

Supplement: Supplementary file 1 — Supplementary Material 1. [file 12672_2025_3715_MOESM1_ESM.jpg]

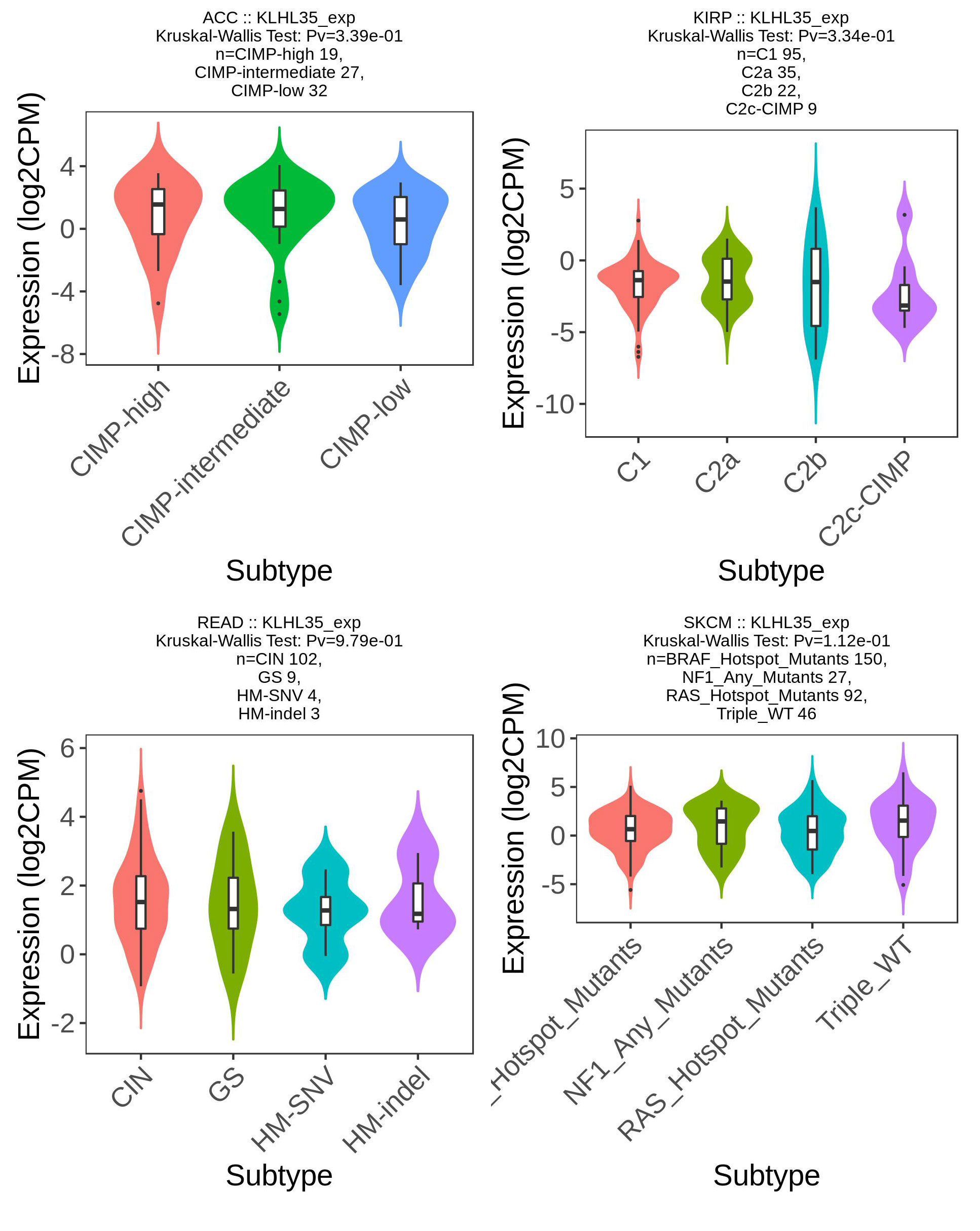

Supplement: Supplementary file 2 — Supplementary Material 2. [file 12672_2025_3715_MOESM2_ESM.jpg]

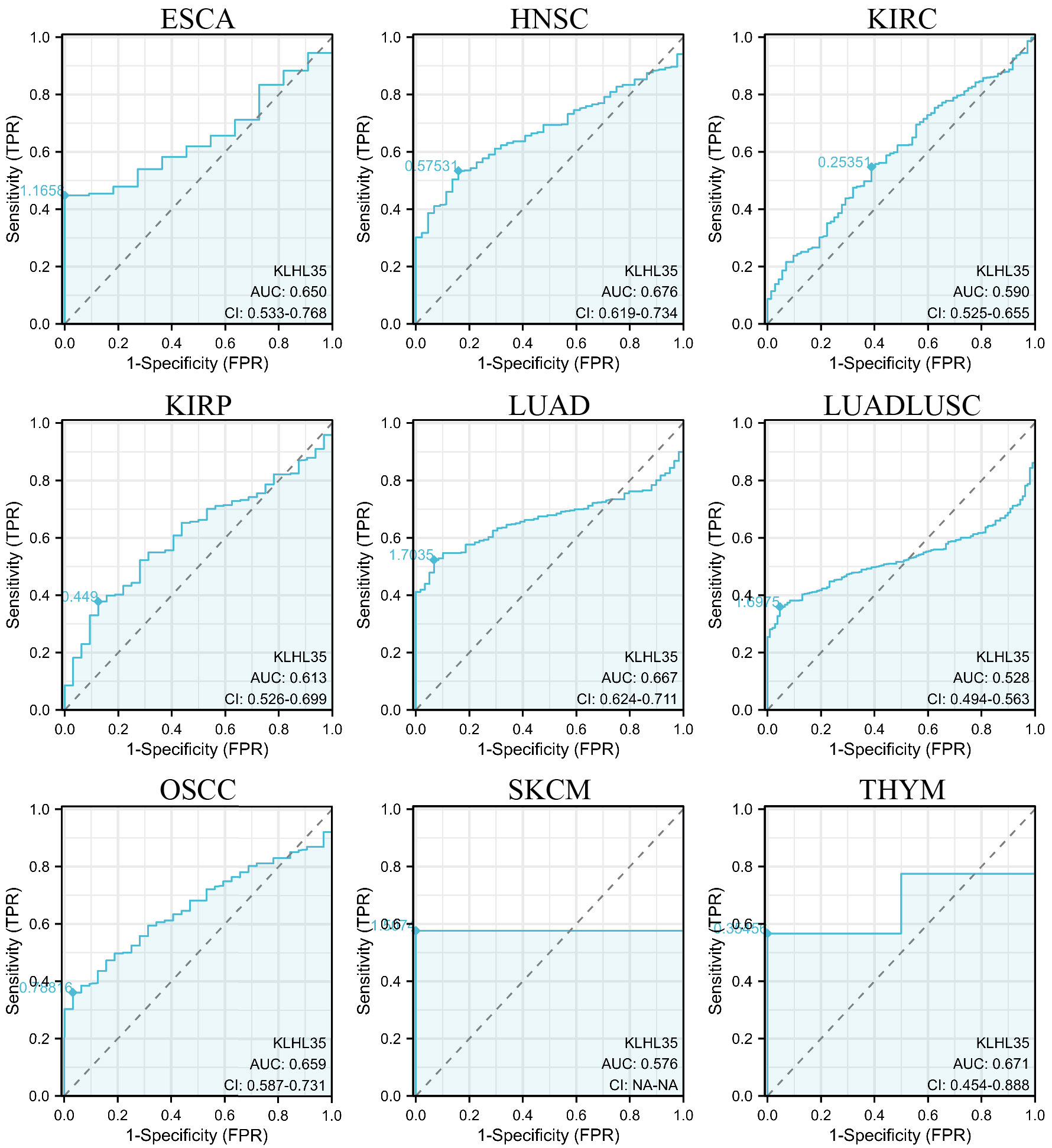

Supplement: Supplementary file 3 — Supplementary Material 3. [file 12672_2025_3715_MOESM3_ESM.jpg]

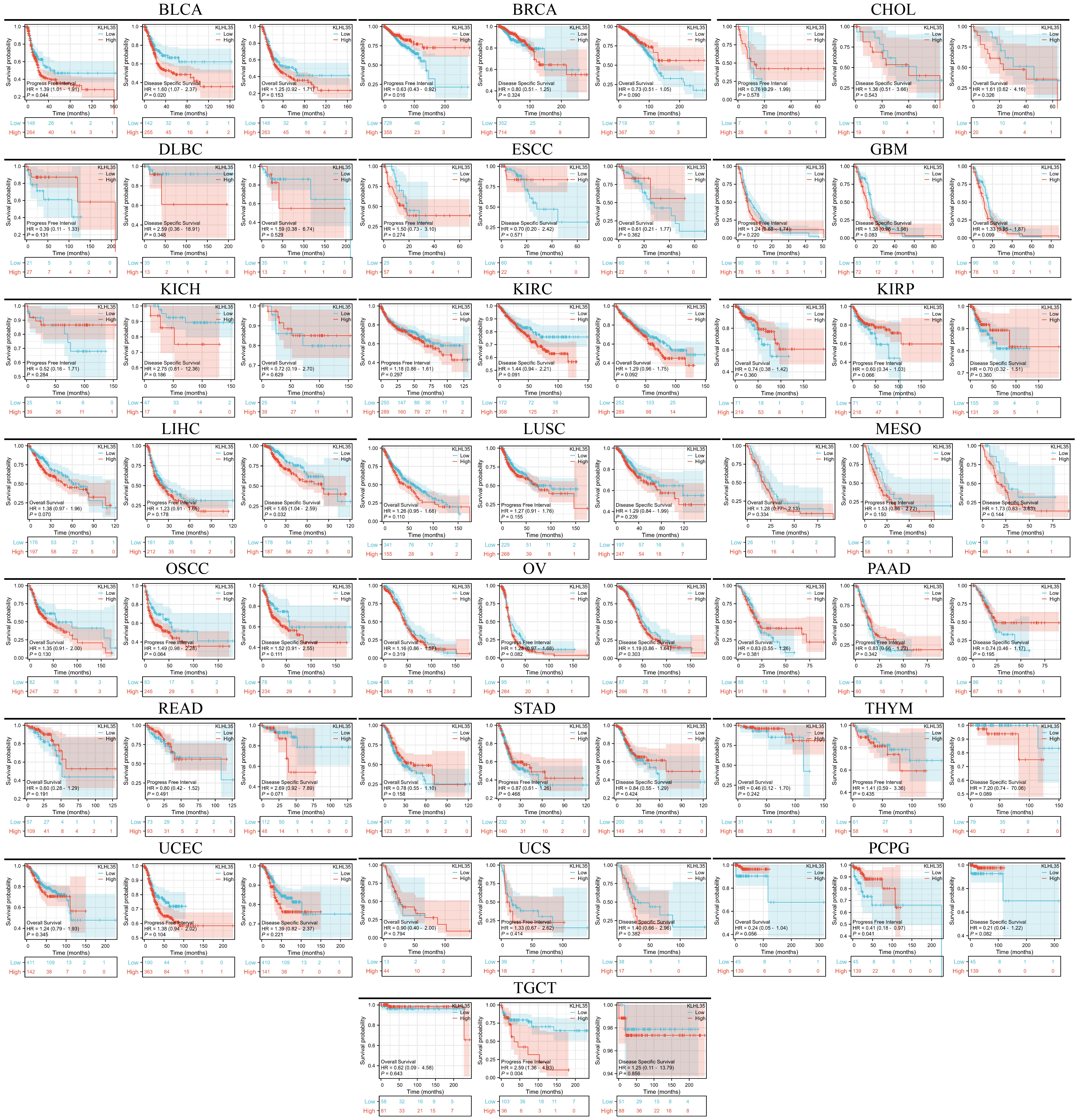

Supplement: Supplementary file 4 — Supplementary Material 4. [file 12672_2025_3715_MOESM4_ESM.jpg]

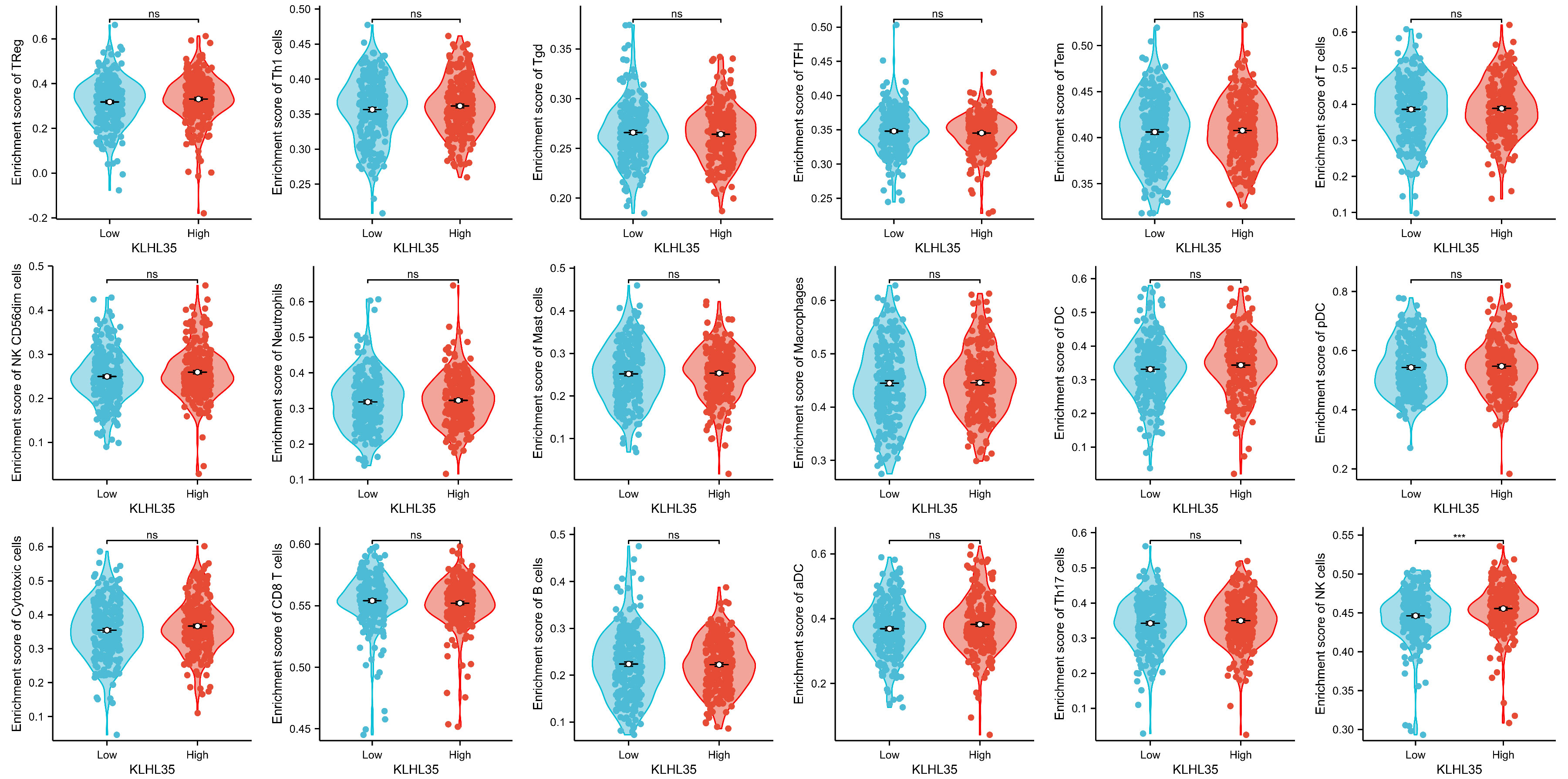

Supplement: Supplementary file 5 — Supplementary Material 5. [file 12672_2025_3715_MOESM5_ESM.jpg]

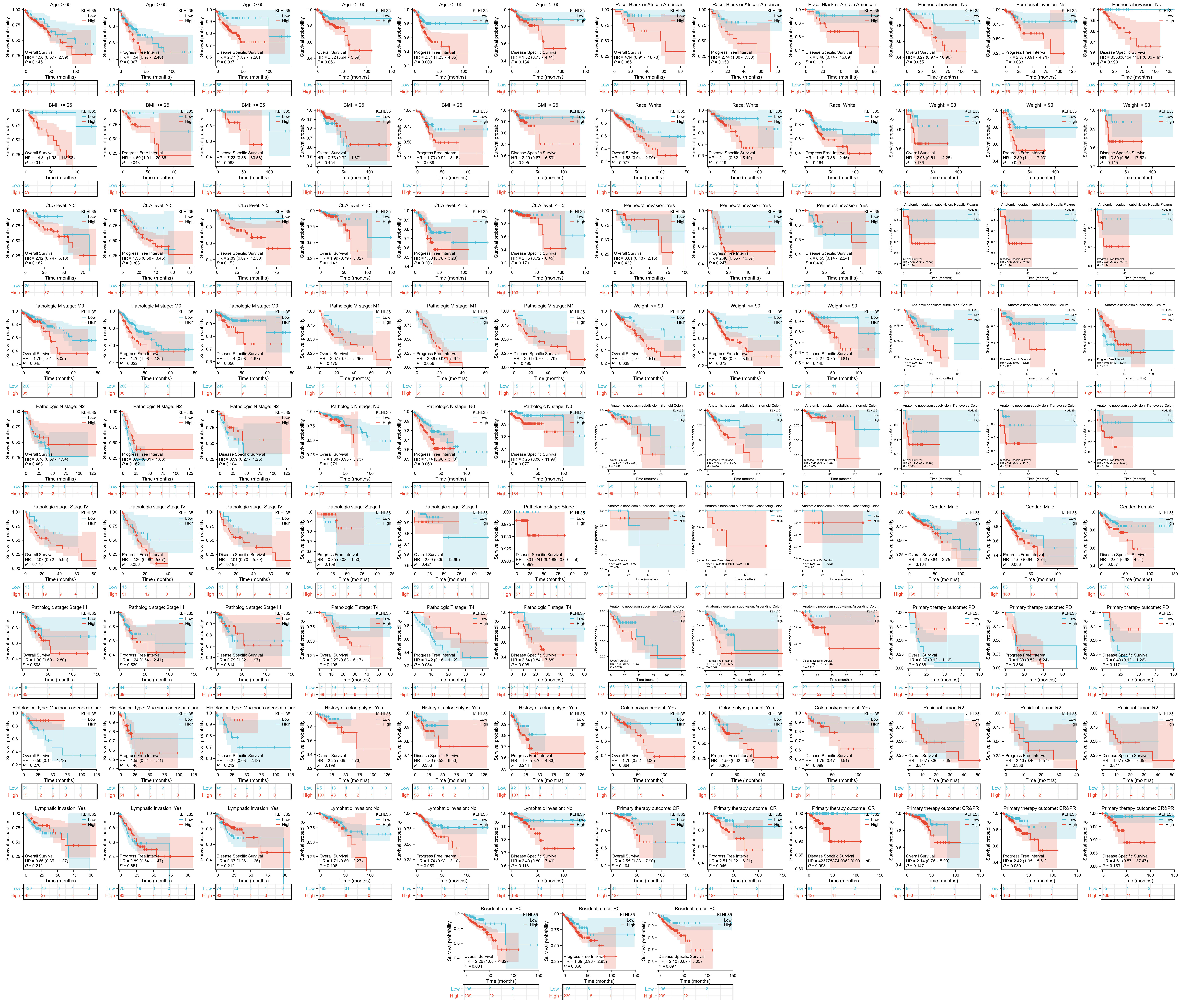

Supplement: Supplementary file 6 — Supplementary Material 6. [file 12672_2025_3715_MOESM6_ESM.jpg]
